# Subjective Cognitive Impairment and Physical Activity: Investigating Risk Factors and Correlations among Older Adults in Spain

**DOI:** 10.3390/jfmk9030150

**Published:** 2024-08-28

**Authors:** Juan Manuel Franco-García, Ángel Denche-Zamorano, Jorge Carlos-Vivas, Antonio Castillo-Paredes, Cristina Mendoza-Holgado, Jorge Pérez-Gómez

**Affiliations:** 1 Health Economy Motricity and Education (HEME) Research Group, Faculty of Sport Sciences, University of Extremadura, 10003 Cáceres, Spain; jmfrancog@unex.es (J.M.F.-G.); jorgepg100@gmail.com (J.P.-G.); 2 Promoting a Healthy Society Research Group (PHeSO), Faculty of Sport Sciences, University of Extremadura, 10003 Cáceres, Spain; denchezamorano@unex.es; 3 Physical Activity for Education, Performance and Health (PAEPH) Research Group, Faculty of Sport Sciences, University of Extremadura, 10003 Cáceres, Spain; jorgecv@unex.es; 4 Grupo AFySE, Investigación en Actividad Física y Salud Escolar, Escuela de Pedagogía en Educación Física, Facultad de Educación, Universidad de Las Américas, Santiago 8370040, Chile; 5 Social Impact and Innovation in Health (InHEALTH), Faculty of nursing and Occupational Therapy, University of Extremadura, 10003 Cáceres, Spain; cristinamh@unex.es

**Keywords:** body composition, dual task, executive function, exercise, memory

## Abstract

Subjective cognitive impairment in older persons has a substantial influence on their quality of life and can progress to serious illnesses such as dementia. Physical activity level can help prevent cognitive decline and improve cognitive performance. The aim of this study was to investigate the association between frequency of physical activity and subjective cognitive impairment in Spanish adults aged 65 and over, and to identify different risk factors. Using data from the EHSS20 survey, the study focused on 7082 participants who provided information on cognitive impairment and physical activity. Key predictor variables included age, gender, BMI, marital status, and education level. A significant relationship was found between BMI category and gender, with 66.5% of the population being overweight or obese. Men were more likely to be overweight than women. Socio-demographic factors such as educational level, marital status, and physical activity frequency showed dependent associations with sex. Women had a higher prevalence of subjective cognitive impairment than men. A strong association was found between frequency of physical activity and subjective cognitive impairment, with inactive older people having the highest prevalence of subjective cognitive impairment. Older women who engage in little physical exercise and have less education are at risk for subjective cognitive impairment. Furthermore, for both men and women, being overweight was associated with a more reduced risk than obesity. Significant relationships were also discovered between subjective cognitive impairment, frequency of physical exercise, gender, BMI, and degree of education. In conclusion, older, sedentary women with high BMI and less education are more likely to experience subjective cognitive impairment.

## 1. Introduction

Subjective cognitive impairment in older people has received much attention because of its impact on quality of life [1]. As the world’s population ages, understanding the impact of subjective cognitive limitations becomes increasingly important to promote well-being and address age-related problems [2]. Subjective cognitive impairment is defined as self-perceived impairments in memory and other cognitive abilities and is thought to be an early indicator of a more serious cognitive impairment, such as moderate cognitive impairment and dementia [3]. Due to their subjective characteristics, diagnostic methods are not entirely clear-cut, and a personalized diagnostic process is recommended to identify or exclude medical conditions [4]. Some of the most commonly used diagnostic strategies are clinical judgement, interviews, psychometric tests such as the Mini-Mental State Examination (MMSE) or the Montreal Cognitive Assessment (MoCA), neuroimaging (such as magnetic resonance imaging and positron emission tomography), and biomarkers (such as amyloid and tau protein levels and olfactory identification) [4,5].

Research has shown that cognitive impairment has important implications for health promotion, [6,7] so understanding cognitive impairment, its incidence, and impact may be fundamental to developing effective interventions to reduce prevalence [8]. In Spain, 4624 individuals in five communities were studied; the adjusted prevalence for the population studied was 18.5%, with women having significantly higher adjusted rates than men, and the prevalence increased exponentially with age, reaching 45.3% in those aged over 85 years [9].

From the perspective of physical and cognitive function, physical activity level (PAL) appears to be an emerging factor that may act as a protective strategy against cognitive impairment [10,11]. Moderate to vigorous physical activity (PA) has been shown to improve cognitive function [12]. Thus, promoting an active lifestyle may help older people maintain their cognitive abilities [13]. However, the frequency of physical activity (PAF) affects the overall effectiveness of PAL [14,15]. Scientific evidence suggests that regular PA is associated with a variety of health benefits, including the prevention and treatment of non-communicable diseases such as heart disease, hypertension, stroke, diabetes, and some malignancies [16]. In particular, PAF has a significant impact on cognitive function, suggesting that regular PA is associated with improved cognitive performance and overall brain health [17,18]. Various types of PA, such as strength training or aerobics (playing tennis, swimming, walking, hiking, or dancing) as well as their intensity and frequency, have an essential role in promoting active ageing, protecting psychophysical well-being, and sustaining cognitive functioning in older persons [19,20,21,22]. In terms of frequency and intensity, compared to sedentary adults, performing PA once a week, one to three times a week, or more, with intensities ranging from low to high, was associated with better cognitive test scores [23]. In regards to time, those who did at least 3 h of PA each week scored considerably higher on the Montreal Cognitive Assessment (MoCA) than those who did less [24]. However, the World Health Organization’s physical activity guidelines recommend for older adults that a minimum of 150–300 min of moderate-intensity physical activity or 75–150 min of vigorous-intensity physical activity weekly (or an equivalent combination of both) are sufficient for health benefits such as improvements in cognitive health [25]. Thus, persistent PA may be required to preserve cognitive function and promote overall well-being in older persons [26,27].

The association between several risk factors and cognitive impairment in older people has also been investigated [28,29]. One of these factors is body mass index (BMI), overweight, and obesity, which have been reported to be more associated with a lower risk of cognitive decline than normal weight in middle-aged and older people [29,30]. However, the role of exercise in this association is critical, as vigorous PA was found to be a mediator, accounting for approximately 5.94% of the association between obesity and cognitive impairment [31]. These findings illustrate the complex relationship between BMI, physical activity, and cognitive health.

Another risk factor is that educational attainment is strongly associated with cognitive impairment in older people, with higher levels of education corresponding to a lower likelihood of dementia and mild cognitive impairment [32,33]. This protective effect may be due to the cognitive reserve, defined as individual differences in the ability to cope with pathological changes in the brain [34] through formal education, as well as healthy lifestyles and access to medical care, which are often associated with higher levels of education [35].

A study conducted in Spain to investigate the prevalence of cognitive impairment in an ageing older population and its association with social factors found a prevalence of 22.2% when distributed by age or level of education, with women having a higher likelihood of cognitive impairment than men [36].

Marital status is also an important factor to consider when determining cognitive impairment [37,38]. The impact of marital status on cognitive functioning in older people has been highlighted by the finding that marital status significantly predicts cognitive impairment [39,40]. Married older people had better cognitive functioning than their single, divorced, or bereaved counterparts [41,42,43]. These findings highlight the need to understand the complexity of social relationships. In Spain, both the incidence of cognitive impairment and marital status are strongly associated with older people’s quality of life and mental health [9,44]. Understanding how marital status affects cognitive function in this population is crucial for the development of effective therapies.

In Spain, the European Health Survey (EHSS20) is one of the valid data sources that can be used to study the association between physical activity frequency and subjective cognitive impairment as well as to identify possible risk factors [45].

Based on the above, it seems crucial to study the associations between subjective cognitive impairment and PAF in the Spanish elderly population, as well as to study risk factors such as age, sex, BMI, and level of education and their association with cognitive impairment, as this may be crucial to better understand the elements influencing mental health and to implement effective intervention strategies to maintain optimal cognitive function. Therefore, the aim of this study was to investigate the association between PAF and cognitive impairment (memory or concentration problems) in people aged 65 years and older in Spain through the EHSS20. We also sought to identify risk factors such as age, sex, physical inactivity, BMI, and educational level within these groups that could explain the prevalence of cognitive deficits.

## 2. Materials and Methods

Data from EHSS20 [45] were used to conduct a cross-sectional descriptive study based on responses. This survey carried out by the National Statistics Institute (INE) and coordinated by the European Statistical Office (Eurostat) aims to investigate the health status of the resident population in Spain, as well as other health markers and socio-demographic factors. The study used a stratified random sampling approach in three stages. First, municipalities were divided into strata and randomly selected. Households in these strata were then randomly selected. Finally, an adult was randomly selected from the selected households. The survey methodology includes all methodological parameters and survey specific information (EHSS20) [45]: data storage and processing, sample calculation, treatment of missing data, how the interviews were conducted, among others.

In accordance with Regulation 2016/679 of the European Parliament and of the Council of the European Union of 27 April 2016 on the protection of individuals with regard to the processing of personal data and on the free movement of personal data, and derogating from Directive 95/46/EC, these data are public and anonymous, and are therefore considered non-confidential data, and data protection principles were not required. No approval from an approved ethics committee was required.

### 2.1. Participants

**Figure 1:**
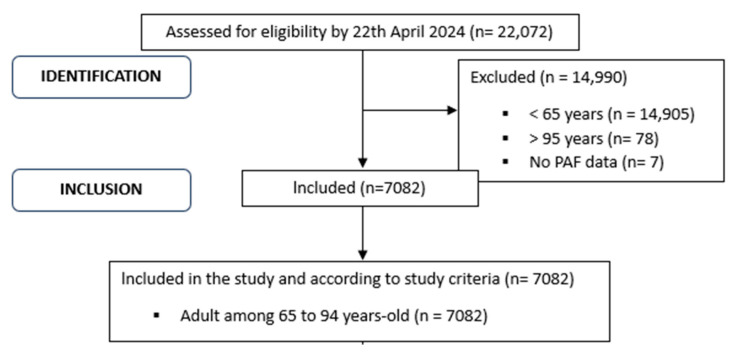
Flow diagram of the study sample’s eligibility criteria.

People who volunteered to participate in the survey were questioned in person by INE-trained staff. It was carried out from July 2019 to July 2020 (due to the COVID-19 pandemic, personal surveys have been conducted by telephone since 17 March 2020). All data and questionnaire responses can be downloaded for free from the INE website. On the website, the data are presented in different formats: .R, .sav, .csv, .sas, and .dta. Therefore, the data can be processed with different statistical programs. For this research, the data were extracted in .sav format (SPSS Statistics Data Document). The EHSS20 had a final sample size of 22,072. All participants were individuals aged 15 and up who lived in family homes in Spain. To get to the final sample of this study, the following inclusion criteria were used: (1) be an advanced adult (aged 65–94); (2) provide data on cognitive impairment (based on response to item Q.38.a: Do you have difficulty to remember or to concentrate?); and (3) provide data on PAF (based on response to item Q.112: Which of these scenarios best characterizes the frequency with which you engage in physical activity in your spare time?). After applying these criteria, 14,990 people were not included (14,905 under 65, 78 over 95, and 7 who did not provide PAF data (they presented “Don’t Know/don’t answer” as a response to item Q.112)), resulting in a final sample of 7082 people. Figure 1 depicts the flow diagram along with the sample’s selection criteria.

### 2.2. Variables Extracted from the Survey

#### 2.2.1. Outcome Variables

Subjective Cognitive Impairment Levels. This variable was extracted from responses to the Q.38.a variable: Do you have difficulty to remember or to concentrate? There are 4 possible answers: (1) No, no difficulty (“None”); (2) Yes, some difficulty (“Some”); (3) Yes, many difficulties (“A lot”); and (4) I can’t do it at all (“Absolutely”), or Don’t Know/don’t answer (DK/DA). Therefore, this variable grouped participants according to these levels of subjective cognitive limitations: “None”, “Some”, “A lot”, and “Absolutely”.

Subjective Cognitive Impairment. This dichotomous variable was created from the responses on the subjective cognitive impairment levels variable (item Q.38.a) and grouped participants into two groups: with subjective cognitive impairment (Yes) and without subjective cognitive impairment (No). For this purpose, the results were grouped into 2 categories: (1) No: those participants who answered (No, no difficulty); (2) Yes: those participants who answered (Yes, some difficulty or yes, many difficulties or can’t do it at all).

#### 2.2.2. Independent, Predictor, and Covariate Variables

Participants who did not provide information on any of the following criteria were excluded from analyses using the variable “no data” but were included in all other analyses.

Age: This continuous variable was obtained from the survey variable “AGEa”.

Sex: This was obtained from the survey variable “SEXOa”, which had two possible responses: men or women.

Physical Activity Frequency (PAF): This is taken from item Q.112. The question was: “Which of these scenarios best characterizes the frequency with which you engage in physical activity in your spare time?” with four possible answers. For this investigation, the groups were called: (1) Never: those individuals who said (I do not exercise); (2) Occasional: individuals who responded (I do occasional physical activity or sport); (3) Frequently: participants who responded (I do physical activity several times a month); and (4) Very frequently: participants who responded (I do physical or sport training several times a week). Or DK/DA.

Body Mass Index (BMI) Group: This was based on the survey variable “BMIa”. Participants were divided into groups based on their BMI (weight in kg divided by height in meters squared). The following four groups were formed: underweight (BMI < 18.5), normal (BMI ≥ 18.5 and <25), overweight (BMI ≥ 25 and <30), and obesity (BMI ≥ 30). A total of 655 participants did not submit data on this variable.

Civil status: This was based on the answers participants provided to item Q.4b: What is your legal marital status? There were five possible answers: (1) Single; (2) Married; (3) Widowed; (4) Legally separated; and (5) Divorced; or (DK/DA). Sixteen participants did not submit data on this variable.

Study level: This information was taken from the “STUDY” (EHSS 2020). These factors represented the greatest level of study attained by the subjects. For this investigation, individuals were divided into five groups: (1) Primary Studies (participants with completed or incomplete primary education); (2) Secondary Studies (participants with compulsory secondary education with or without a diploma); (3) Bachelor’s Degree (participants with Bachelor’s Degree Studies); (4) Vocational Training (participants with Vocational Education and Training at an intermediate or higher level or equivalent); and (5) University.

### 2.3. Statistical Analysis

The Kolmogorov–Smirnov test was used to determine the normality of the data for the continuous variable (age). Descriptive analysis was used to characterize the sample using the following variables: age (median and IQR, a continuous variable), BMI group, Study Level, Civil Status, PAF, Subjective Cognitive Impairment, and Subjective Cognitive Impairment Levels (absolute and relative frequencies, categorical variables). The Mann–Whitney U test was used to examine possible age differences between participants by gender. The Chi-square test was used to examine potential dependent relationships between sex and all categorical variables. Cramer’s V and Phi coefficients were calculated where appropriate to determine the strength of these associations. The post hoc paired z-test for independent proportions was used to investigate potential sex differences in the proportions of categorical variables.

The Chi-square test was used to examine the dependent associations between PAF and the variables Subjective Cognitive Impairment and Subjective Cognitive Impairment Levels. Cramer’s V coefficient was used to determine the strength of the associations. To investigate potential differences in the proportions of subjective cognitive impairment and subjective cognitive impairment levels as a function of PAF, the post hoc paired z-test for independent proportions was used.

Multiple binary logistic regression was used to examine the risks of having subjective cognitive impairment, with subjective cognitive impairment as the dependent variable and the remaining study factors (Age, Sex, BMI group, Civil Status, Study Level, and PAF) as independent variables. A significance level greater than 0.95 was used for all analyses. All analyses were performed using the statistical program IBM SPSS Statistical version 25.3.

## 3. Results

The statistics on the participants’ ages were not regularly distributed (*p* < 0.001). The sample median age was 75 (12) years, with no sex differences (*p* = 0.129). BMI category and sex showed a dependent connection (X^2^ = 85.2, df = 3, *p* < 0.001, V = 0.115). In total, 66.5% of the population was considered overweight or obese. Men were more likely to be overweight than women (52.3% vs. 41.5%, *p* < 0.001). Dependence correlations were established between sex and various socio-demographic factors such as Study Level (X^2^ = 107.4, df = 4, *p* < 0.001, V = 0.123), Civil Status (X^2^ = 851.7, df = 4, *p* < 0.001, V = 0.347), and PAF (X^2^ = 97.9, df = 3, *p* < 0.001, V = 0.118). Subjective memory impairments were shown to be associated with sex (X^2^ = 73.6, df = 3, *p* < 0.001, V = 0.102) and prevalence (X^2^ = 71.3, df = 1, *p* < 0.001, φ = 0.100). Women exhibited a greater prevalence of subjective cognitive impairment compared to men (31.2% vs. 22.1%, *p* < 0.001) (Table 1).

**Figure 2:**
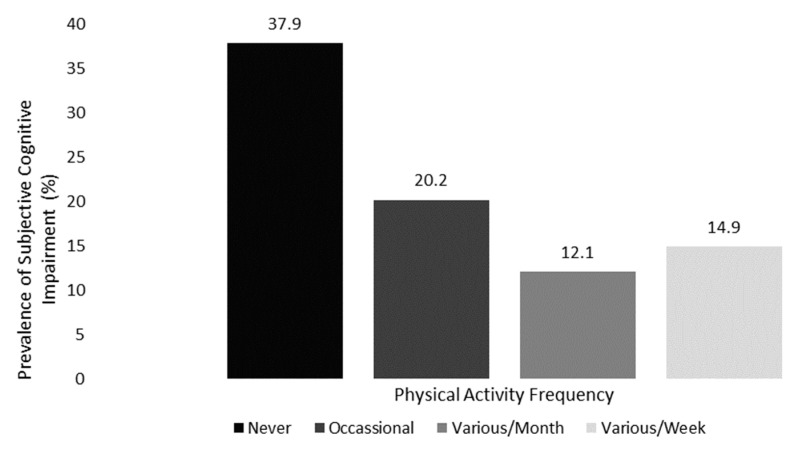
Prevalence of subjective cognitive impairment according to PAF.

A dependency association was discovered between PAF and subjective cognitive impairment (X^2^ = 345.9, df = 3, *p* < 0.001, V = 0.221) (Supplementary Materials Table S1). The inactive elderly had the highest prevalence of subjective cognitive impairment (37.9%), with significant differences from the other categories (Supplementary Materials Table S1). Figure 2 depicts the prevalence of subjective cognitive impairment as a function of PAF.

PAF and subjective cognitive impairment levels showed a dependent connection (X^2^ = 437.3, df = 9, *p* < 0.001, V = 0.143). In all the subjective cognitive impairment levels, the inactive groups had a higher prevalence, with the lowest prevalence seen in the groups that exercised more regularly, with significant differences between these group (Table 2).

In analyzing risk factors for subjective cognitive impairment, women had a higher risk than men (OR: 1.25, CI95%: 1.11–1.42, *p* < 0.001), as did older age (OR: 1.08, CI95%: 1.07–1.09, *p* < 0.001). In contrast, those with a greater PAF and educational level were at a decreased risk (Table 3). The overweight group had the lowest risk of subjective cognitive impairment (OR: 0.84, CI95%: 0.72–0.98, *p* < 0.001) compared to the obese group, whereas there were no significant differences in risk among the other groups. Female, elderly, inactive, with a low educational level, and obesity are the characteristics associated with the highest chance of suffering from subjective cognitive impairment. The model accounted for 17% of the variance (Nagelkerke’s R^2^).

## 4. Discussion

This study investigated the relationships between subjective cognitive impairment (difficulty remembering or concentrating) and PAF in people aged 65 years old and over in Spain. The risks factors associated with subjective cognitive impairment (sex, BMI, level of education, marital status) were also examined. The risk profile of subjective cognitive impairment was also determined. The main results showed dependent correlations between subjective cognitive impairment and PAF. Significant relationships were also found between these limitations and risk factors such as sex, BMI, and level of study. It was also found that being an older, sedentary woman with a low level of education and a BMI above 30 put you at the highest risk of developing subjective cognitive impairment.

PA is therefore considered one of the most important modifying and preventive agents, as previous research has shown that modifiable risk factors are determinants of the onset of cognitive decline, including subjective memory complaints, mild cognitive impairment, or dementia [46]. There is evidence of a strong relationship between subjective cognitive impairment and PAF [23,47,48]. We found that older people with a higher PAF had a significant reduction in subjective cognitive impairment over time, compared to those who were less physically active [49]. Other findings consistent with ours suggest that regular PA may have a protective effect against perceived cognitive impairment [50]. Furthermore, subjective memory complaints are a crucial stage in the development of preventive therapeutic strategies to prevent the further development of a pathological clinical state [51]. Despite the data presented above, it may be important to conduct additional research on the relationship between PAF and perceived cognitive impairment, as it is more complex than previously thought and may be mediated by other factors such as general health status and psychosocial factors [52].

BMI, as an indicator of overall health [53], can be significantly influenced by regular physical activity, which not only helps to maintain a healthy BMI by burning calories but also improves body composition and regulates metabolism. This suggests that physical activity is essential for managing and preventing health problems associated with high BMI [54]. Longitudinal studies have shown that those who exercise regularly are less likely to develop obesity [55,56,57]. In addition, a high BMI, particularly in obese people, has been associated with an increased likelihood of subjective cognitive impairment [58,59]. This association has been attributed to a variety of causes, including the detrimental effects of increased body fat on vascular and metabolic health [29,60]. Obesity is associated with chronic inflammation, insulin resistance, and endothelial dysfunction, all of which can lead to cognitive impairment [61]. Regular PA has been associated with significant improvements in memory and other cognitive abilities in older people, particularly those at higher risk of cognitive decline due to high BMI [62,63]. Scientific research suggests that promoting active lifestyles may be an effective strategy for improving metabolic and cognitive health, particularly in at-risk populations [64]. In addition to being active and healthy, non-modifiable characteristics such as age, sex, and education have been found to explain some of the differences in cognition in older adults [65].

Educational attainment as a non-modifiable aspect, together with Stern’s contributions on cognitive reserve, has found potential applications in the field of healthy ageing [66]. Thus, aspects such as educational attainment and other lifestyle-related aspects have been studied extensively. These studies have shown that a higher level of education increases the set of skills and personal characteristics that enable the person to cope better with brain damage and cognitive decline, both in healthy ageing and in the onset of cognitive disorders such as mild cognitive impairment [67]. It is therefore necessary to consider cognitive reserve as a protective factor in cognitive ageing. In the analysis of our results, the data are consistent with this trend and show an association between academic level and cognitive complaints. However, other studies have found that the correlation between educational and global cognitive change is unrelated [68]. One hypothesis that could address this ambiguity is the difficulty in finding measurable and objective tools to assess the amount of cognitive reserve and the appropriate way to apply these indicators in research [69].

With regard to sex, our findings are similar to those of previous research, particularly in pathological conditions, where greater cognitive impairment is observed in women in comparison to men [70]. However, it could be argued that there are several factors conditioning this phenomenon. Firstly, age is the main non-modifiable risk factor for the occurrence of cognitive impairment, so it should be noted that women have a longer life expectancy, which may determine a higher prevalence in the female gender [71]. However, in our data analysis, the median age of the women is higher than that of men, but this difference is not statistically significant, so this hypothesis is not consistent with our results. Second, as other studies have shown, subjective memory complaints such as those reflected in our research, can also be attributed to depressive states and psychiatric comorbidities [51,72]. This observation is noteworthy because previous studies have found a positive association between various factors associated with geriatric depression, such as female gender or low daily physical activity [73]. PA has also shown anti-depressant benefits and may be useful for public health interventions [74].

Socio-cultural factors may also play a role in the development and maintenance of cognitive impairment in older people [75]. Historically, women have had less access to culture and formal education in recent decades, and this is reflected in our results. In our sample, we observed that women outnumber men only at the primary level of education; the higher the level of education, the lower the presence of women, and the median is lower than that of men. This difference is significant at the secondary education, high school, and university levels. As discussed earlier, an individual’s educational background is a component of cognitive reserve. Therefore, individuals with a better cognitive reserve are likely to be better able to cope with the changes brought about by cognitive ageing [76].

Based on our expertise, the robustness of our findings and the representativeness of the sample considered indicate considerable potential. However, the study had some limitations. Due to the cross-sectional nature of study, causal relationships between PAF and cognitive impairment could not be established. Participants rated their cognitive impairment and frequency of physical activity in the EHSS using self-report questionnaires. In the case of cognitive performance, there may be a bias in people who have cognitive impairment but experience anosognosia in the early stages and are unable to perceive the impairment. Therefore, this survey should include tests that assess participants’ cognitive abilities as well as objective data on people’s PAF. It would be useful to have both objective and subjective data to characterize the variables in this group. In addition, on the 17 March 2020, the confinement in Spain was declared. From this date onwards, interviews were conducted by telephone rather than in person, which may have influenced the results. The limitations of this study provide an opportunity for future research. It is also important to note that although BMI is widely used to assess weight status, there are many factors that need to be considered [77]. BMI does not distinguish between people with low skeletal muscle mass, high fat weight, sarcopenic obesity, and normally healthy people with higher skeletal muscle mass and lower fat weight, nor does it determine how fat mass is distributed; it may also vary by gender, race, ethnicity, or age [77]. Therefore, body composition data would need to be complemented by other measures such as waist-to-hip ratio [78,79].

Based on the results of this study, and taking into account the limitations mentioned above, the availability of these data could help promote community health strategies and implement prevention programs aimed at reducing physical inactivity and inactive behaviors to improve the cognitive health of the elderly Spanish population. The researchers believe that maintaining an adequate level of physical activity at this stage of life may protect against age-related cognitive impairment. In addition, the factors that influence subjective cognitive impairment and decline in older people need to be identified and taken into account in order to improve approaches to preventing cognitive decline and dementia.

Future studies should further investigate the relationship between PAF and subjective cognitive impairment, considering the complexity of the relationship and the likelihood that it is mediated by other factors, such as general health status and psychosocial variables. In addition, longitudinal research would be beneficial to establish causal relationships and use objective instruments to assess both cognitive function and physical activity. Additional measures of body composition, such as waist-to-hip ratio, need to be included to complement BMI assessments and provide a more complete understanding of the impact of obesity and other factors on cognitive health. Future studies should also look at gender differences and the role of cognitive reserve in preventing cognitive decline, considering the impact of socio-cultural and educational factors on these dynamics.

## 5. Conclusions

We conclude that there are significant associations between subjective cognitive impairment and frequency of physical activity in Spanish adults aged 65 and over. Thus, the frequency of physical activity was identified as a potential risk factor for subjective cognitive impairment, together with sex, BMI, and educational level. The lowest risk of self-reported cognitive impairment was found in older people with a frequency of several times per month. People who exercised several times a month had less than a third of the risk reported by inactive people, while those who exercised several times a week had half the risk. In addition, it was revealed that being an older, inactive woman, and having a BMI greater than 30 and a low level of education were the highest risk profiles for subjective cognitive impairment in Spain.

## Figures and Tables

**Table 1:** Descriptive analysis.

Variables	Total = 7082		Men = 2994		Women = 4088		X^2^	df	*p*	V
	Median	IQR	Median	IQR	Median	IQR				
Age (Years)	75	(12)	74	(11)	76	(13)	n.a.	n.a.	0.129	n.a.
BMI Group	n	%	n	%	n	%	X^2^	df	*p* *	V
Underweight	77	1.2	21	0.7	56	1.6 **	85.2	3	<0.001	0.115
Normal	2073	32.3	774	27.5	1299	36.0 ***				
Overweight	2968	46.3	1471	52.3	1497	41.5 ***				
Obesity	1299	20.2	547	19.4	752	20.9				
Study Level										
Primary	4250	60.0	1628	54.4	2622	64.1 ***	107.4	4	<0.001	0.123
Secondary	1221	17.2	510	17.0	711	17.4				
Bachelor’s Degree	550	7.8	280	9.4	270	6.6 ***				
Vocational training	359	5.1	191	6.4	168	4.1 ***				
University	702	9.9	385	12.9	317	7.8 ***				
Civil Status										
Single	612	8.7	322	10.8	290	7.1 ***	851.7	4	<0.001	0.347
Married	3584	50.7	2006	67.1	1578	38.7 ***				
Widowed	2486	35.2	478	16.0	2008	49.3 ***				
Legally separated	165	2.3	88	2.9	77	1.9 **				
Divorced	219	3.1	95	3.2	124	3.0				
PAF										
Inactive	3222	45.5	1159	38.7	2603	50.5 ***	97.9	3	<0.001	0.118
Occasional	2903	41.0	1396	46.6	1507	36.9 ***				
Various/Month	405	5.7	183	6.1	222	5.4				
Various/Week	552	7.8	256	8.6	296	7.2 *				
Subjective Cognitive Impairment Levels										
No	5146	72.7	2332	77.9	2814	68.8 ***	73.6	3	<0.001	0.102
Yes, something	1483	20.9	521	17.4	962	23.5 ***				
Yes, a lot	345	4.9	108	3.6	237	5.8 ***				
Yes, absolutely	108	1.5	33	1.1	75	1.8 *				
Subjective Cognitive Impairment							X^2^	df	*p*	φ
No	5146	72.7	2332	77.9	2814	68.8 ***	71.3	1	<0.001	0.100
Yes	1936	27.3	662	22.1	1274	31.2 ***				

df (degree freedom); IQR (Interquartile range); n (participants); n.a. (not applicable); % (Percentage); *p* (*p*-value from Mann–Whitney U test); *p* * (*p*-value from Chi-square test); * (significant differences in proportions between gender with *p* < 0.05 (** *p* < 0.01; *** *p* < 0.001) from post hoc pairwise z-test for independent proportions); X^2^ (Chi-square statistic); V (Cramer’s V coefficients); φ (Phi coefficients).

**Table 2:** Subjective cognitive impairment levels according to PAF.

Variables	Physical Activity Frequency								X^2^	df	*p*	V
Subjective Cognitive Impairment Levels	Never (A)		Occasionally (B)		Frequently (C)		Very Frequently (D)					
	n	%	n	%	n	%	n	%				
None	2002	61.1%	2318	79.8%	356	87.9%	470	85.1%	437.3	9	<0.001	0.143
Some	848	26.3%	563	17.9%	45	11.1%	71	12.9%				
A lot	275	8.5%	56	1.9%	4	1.0%	10	1.8%				
Absolutely	9	3.0%	10	0.3%	0	0.0%	1	0.2%				
Proportions’ differences post hoc												
None				A (*p* < 0.001) ***		A (*p* < 0.001) *** B (*p* = 0.001) **		A (*p* < 0.001) *** (*p* = 0.023) *				
Some		B (*p* < 0.001) *** C (*p* < 0.001) *** D (*p* < 0.001) ***		C (*p* = 0.004) ** D (*p* < 0.025) *								
A lot		B (*p* < 0.001) *** C (*p* < 0.001) *** D (*p* < 0.001) ***										
Absolutely		B (*p* < 0.001) *** D (*p* = 0.001) ***										

df (degree freedom); *p* (*p*-value from pairwise z-test for independent proportions); * (*p* < 0.05); ** (*p* < 0.01); *** (*p* < 0.001); X^2^ (Chi-Square); V (V‘s Cramer coefficients).

**Table 3:** Logistic binary regression model for memory problems risk factor.

Model for Subjective Cognitive Impairment					
	β	Adjusted OR	95% C.I.		*p*-Value
Age	0.073	1.08	1.07	1.09	<0.001 ***
Sex					
Men		Reference			
Women	0.224	1.25	1.11	1.42	<0.001 ***
PAF					
Never		Reference			<0.001 ***
Occasional	−0.519	0.60	0.52	0.68	<0.001 ***
Various/Month	−1.152	0.32	0.22	0.45	<0.001 ***
Various/Week	−0.652	0.52	0.40	0.68	<0.001 ***
Study Level					
Primary		Reference			<0.001 ***
Secondary	−0.381	0.68	0.57	0.81	<0.001 ***
Bachelor’s Degree	−0.771	0.46	0.35	0.61	<0.001 ***
Vocational Training	−0.289	0.75	0.55	1.01	0.060
University	−0.786	0.46	0.36	0.58	<0.001 ***
BMI Group					
Obesity		Reference			0.029 *
Underweight	−0.003	1.00	0.59	1.68	0.991
Normal	0.018	1.02	0.86	1.21	0.833
Overweight	−0.175	0.84	0.72	0.98	0.030 *
Constant	−6.261	0.00			<0.001 ***

β (Beta); CI (Confidence Interval).; OR (Odds ratio); * (*p*-value < 0.05); *** (*p*-value < 0.001).

## Data Availability

Data will be made available upon reasonable request by the corresponding author.

## Supplementary Materials

The following supporting information can be downloaded at: https://www.mdpi.com/article/10.3390/jfmk9030150/s1. Table S1. Subjective Cognitive Impairment according to Physical Activity Frequency.
