# Supplementary material for: Subjective Cognitive Impairment and Physical Activity: Investigating Risk Factors and Correlations among Older Adults in Spain
Source: J Funct Morphol Kinesiol. 2024 Aug 28;9(3):150. doi: 10.3390/jfmk9030150 (PMC11417891; doi:10.3390/jfmk9030150)
Supplement: Supplementary file 1 [file jfmk-09-00150-s001.zip › Table S1. Subjective Cognitive Impairment according to Physical Activity Frequency..pdf]

Table S1. Subjective Cognitive Impairment according to Physical Activity Frequency.

Variables	PAF								X^2	df	p	V
Subjective Cognitive Impairment	Never (A)		Occasionally (B)		Frequently (C)		Very Frequently (D)					
	n	%	n	%	n	%	n	%				
No	2002	62.1%	2318	79.8%	356	87.9%	470	85.1%	345.9	3	<0.001	0.221
Yes	1220	37.9%	585	20.2%	49	12.1%	82	14.9%				
Proportions's differences post hoc												
No				A (p<0.001) ***		A (p<0.001) ***<br>B (p=0.001) **		A (p<0.001) ***<br>B (p=0.023) *				
Yes		B (p<0.001) ***<br>C (p<0.001) ***<br>D (p<0.001) ***		C (p=0.001) *<br>D (p=0.023) *								

p (p-value from pairwise z-test for independent proportions); * (p<0.05); ** (p<0.01); *** (p<0.001); X^2 (Chi-Square); df (Degree freedom); V (V's Cramer coefficients).
